# Advances in Gut Microbiome Research, Opening New Strategies to Cope with a Western Lifestyle

**DOI:** 10.3389/fgene.2016.00224

**Published:** 2017-01-10

**Authors:** Gina P. Rodriguez-Castaño, Alejandro Caro-Quintero, Alejandro Reyes, Fernando Lizcano

**Affiliations:** ^1^Center of Biomedical Research, CIBUS, Universidad de La SabanaChía, Colombia; ^2^Corporación de Investigación Agropecuaria CORPOICA, Centro de Investigación TibaitatáMosquera, Colombia; ^3^Department of Biological Sciences, Universidad de los AndesBogotá, Colombia; ^4^Center for Genome Sciences and Systems Biology, Washington University School of MedicineSt. Louis, MO, USA; ^5^Department of Pathology and Immunology, Washington University School of MedicineSt. Louis, MO, USA

**Keywords:** nutrition, microbiome, obesity, lifestyle, inflammation

## Abstract

The “westernization” of global eating and lifestyle habits is associated with the growing rate of chronic diseases, mainly cardiovascular diseases, cancer, type 2 diabetes mellitus, and respiratory diseases. The primary prevention approach is to make nutritional and behavioral changes, however, there is another important determinant of our health that only recently has been considered and is the presence of beneficial microorganisms and their products in our gastrointestinal tract. Microorganisms living in our body can alter the fate of food, drugs, hormones, and xenobiotics, and recent studies point to the use of microorganisms that can counteract the harmful effects of certain compounds introduced or produced endogenously in our body. This review considers the effects of the western lifestyle on adiposity, glucose metabolism, oxidative markers and inflammation profile, emphasizes on the studies that have investigated bacterial strains and products of their metabolism that are beneficial under this lifestyle, and examines the screening strategies that recent studies are using to select the most promising probiotic isolates. In addition, we consider the relevance of studying the microbiota of metabolically healthy people under a western lifestyle for the understanding of the key components that delay the development of chronic diseases.

## Introduction

Our modern societies have settled on large urban arrangements that have changed behavior and alimentary patterns. The establishment of new alimentary habits has been influenced by industrialization and technological advances which have minimized the time for preparing and consuming meals, reduced the cost of livestock, dairy, and sugar-sweetened products, vegetable oils, and flours, and increased the availability of these foods, especially, to low-income families (Drewnowski and Popkin, 1997). Nowadays, the trend in nutritional epidemiology is the analysis of dietary patterns (i.e., through food-frequency questionnaires) to assess habits in food consumption. In this line, several studies have focused on identifying the main dietary factors that are common to the modern diet. For instance, Hu et al. (1999) identified a “western dietary pattern” through factor analysis of dietary patterns among cohorts in the United States. The authors described this dietary pattern as a diet with a “higher intake of processed meat, red meat, butter, high-fat dairy products, eggs, and refined grains”. Likewise, Slattery et al. identified a similar dietary pattern with “high levels of red meat, processed meat, fast food, refined grains, and sugar- containing foods, and low levels of vegetables (other than potatoes) and fruits, with the predominant fruit being canned fruit” (Slattery et al., 1998; Hu et al., 1999). Importantly, the “western diet” is no longer restricted to western societies, globalization and urbanization are increasing the worldwide exposition to this dietary pattern. For example, a Japanese study found a “westernized Japanese pattern” associated with “high intakes of bread, meat, processed meat, fruit juice, coffee, black tea, soft drinks, sauces, mayonnaise, and dressing” (Nanri et al., 2012). Overall, the “western diet” can be understood as a dietary pattern with a high intake of refined sugars, refined vegetable oils, and livestock products, and low intake of fresh fruits and vegetables (Cordain et al., 2005).

Concerns with the modern alimentary pattern can be traced back to the scientific literature of 1939, when Weston Price published his findings on modern degeneration related to the modernized diet (Price, 1939). Currently, not only the modern dietary habits are of concern but also low-activity high-stress occupations, sedentarism, alcohol binge drinking, and smoking. These behavior and alimentary patterns will be defined from now on as the “western lifestyle” (CDC, 2012; Parry and Straker, 2013; WHO, 2014). This lifestyle is increasingly being associated with several conditions, including: obesity, Alzheimer's disease (Kanoski and Davidson, 2011), cardiovascular disease, type 2 diabetes mellitus (Bazzano, 2005), non-alcoholic fatty liver disease (NAFLD) (Trovato et al., 2015), hypertension (Geleijnse et al., 2004), osteoporosis (Jehle et al., 2006), autoimmunity (Manzel et al., 2014), and cancer (Adlercreutz, 1990). There are several risk factors for developing chronic diseases, including genetic, environmental, demographic, social, and other factors that are not the scope of this review, instead the objective of this review is to relate the research made in the fields of microbiology, immunology, and nutrition to explain the role of gut microbiota as a risk factor of “western lifestyle”-related chronic conditions, and then the strategies that are being developed to shift the gut microbiota from a risk factor toward a more protective state that helps ameliorate the effects of this lifestyle.

## The healthy western microbiota

The concept of the human microbiota, as first described by Joshua Lederberg, is defined as “the ecological community of commensal, symbiotic, and pathogenic microorganisms that literally share our body space” (Lederberg and McCray, 2001). Major efforts are being made worldwide in order to understand the composition and functional states of the healthy gut microbiota. So far, projects like the Human Microbiome Project Consortium among others (Eckburg et al., 2005; Qin et al., 2010; Huttenhower et al., 2012) have found that the gut microbiome of healthy individuals varies significantly and only dominant bacterial phyla have been consistently described, these are Firmicutes, Bacteroidetes and Actinobacteria, with Proteobacteria and Verrucomicrobia also present in lower abundance. Other studies also evidence that the microbiome of healthy and non-healthy states can be distinguished, as it is the case for ulcerative colitis, Crohn's disease (Qin et al., 2010), chronic fatigue syndrome (Frémont et al., 2013), rheumatoid arthritis (Zhang et al., 2015), type I diabetes (Brown et al., 2011), and type II diabetes (Larsen et al., 2010); nevertheless, one study warns that the patient's treatment can exert changes in the microbiota (Forslund et al., 2015).

Although not a single marker can be identified as representative of a healthy gut microbiome, a higher proportion of butyrate-producing and mucin-degrading bacteria has been mentioned in some studies (Brown et al., 2011; Joossens et al., 2011; Erickson et al., 2012). Butyrate is a short chain fatty acid produced mainly by bacterial fermentation of non-digestible fiber in the colon, and a correct balance of a butyrate-producing microbiota may induce the synthesis of mucin in the gut epithelium thus maintaining gut integrity (Finnie et al., 1995; Brown et al., 2011). Studies have shown that butyrate can enhance the assembly of tight junction proteins through regulation of AMP-activated protein kinase (AMPK), however the mechanism of AMPK activation is unknown (Peng et al., 2009). Butyrate also has anti-inflammatory and anti-carcinogenesis effects, mainly by two mechanisms: activation of GPCRs (GPR41 and GPR43) and inhibiton of histone deacetylase (HDAC). Some of the effects of butyrate that have been observed are enhancement of the expression of certain pro-apoptotic genes in malignant cells and suppression of the pro-inflammatory pathway of Nuclear Factor kappa beta (NF-κβ) (Vinolo et al., 2011). It is estimated that butyrate producers represent approximately 25% of all human fecal bacteria (Louis et al., 2010). Meanwhile, *Bifidobacteria* is another important group for colon health, they represent about <5% of the microbiota in adult subjects. In disease states like *Clostridium difficile* associated diarrhea, a 3 log_10_ reduction of this group of bacteria can occur (Hopkins et al., 2001). *Bifidobacteria* contributes to colon health through the production of organic acids, like acetate and lactate, that are then used by butyrate-producing bacteria. Thus, a high abundance of butyrate-producers, mucin-degraders, and *Bifidobacteria* could be an indicator of good health.

Another common feature in some studies is greater gut diversity in healthy states. In lean twins, a greater bacterial diversity has been observed compared to their obese twins (Turnbaugh et al., 2009), in patients with morbid obesity subjected to a gastric bypass an increased richness of gut microbiota was also observed after the surgery along with positive health outcomes (Kong et al., 2013), and another study analyzed the microbiota of non-colic and colic infants finding a higher microbiota diversity in non-colic infants during the first weeks after birth (de Weerth et al., 2013). Hence, a high bacterial diversity can be another indicator of a healthy gut microbiota.

In terms of western diet, Yatsunenko et al. observed that American microbiomes were enriched with genes degrading simple sugars and amino acids (Yatsunenko et al., 2012). As mentioned earlier, a western diet is characterized by a higher intake of processed meat and red meat, thus individuals following this diet may benefit from a Bacteroides-rich microbiota instead of a Prevotella-rich microbiota. This last type of microorganisms produces more trimethylamine from L-carnitine, a nutrient in red meat, which is then converted to pro-atherosclerotic trimethylamine-N-oxide (TMAO), increasing the risk of atherosclerosis. A study by Lozupone et al. evidenced that the immune dysfunction of HIV-infected individuals compromises their ability to select for bacteria that match their diet, thus HIV-positive individuals following a western diet, instead of having a Bacteroides-rich microbiota have a Prevotella-rich microbiota, which is normally present in individuals consuming a plant-based diet, in consequence, these HIV-positive subjects have an increased incidence of several health risks, including cardiovascular disease (Koeth et al., 2013; Tang et al., 2013; Lozupone et al., 2014). Therefore, a Bacteroides-rich microbiota is of benefit under a western diet, as it has been associated with reduced cardiovascular risk.

In order to reveal the key components that make a healthy western microbiota, studies at the strain-level are needed. Evidence points that different strains have distinct effects, as it is the case with strains belonging to a genus enriched in people following a western diet, *Lactobacillus* (Armougom et al., 2009; Million et al., 2012; Poutahidis et al., 2013). For example, the administration of *Lactobacillus reuteri* ATCC PTA 4659 was associated with weight decrease in mice, whereas the administration of *L. reuteri* L6798 was associated with weight gain (Fåk and Bäckhed, 2012). Differential effects have also been observed on the type of immunological response that the strain elicits, for example, *Lactobacillus salivarius* CECT5713 induced the anti-inflammatory cytokine IL-10, while *Lactobacillus fermentum* CECT5716 induced pro-inflammatory cytokines (Díaz-Ropero et al., 2007). Studying the intestinal microbiota at the strain-level has been proved challenging due to the great variability at this taxonomic level among individuals and the lack of reliable, easy to use tools for accurate identification of bacteria at strain level, however, these studies indicate that the insights obtained at the phylum-level are limited and that the understanding of the functionality of strains can help delineate the boundaries of a healthy gut microbiota.

### Metabolic healthy subjects under a western lifestyle

In order to better understand the healthy western microbiota, metabolic healthy individuals following a western lifestyle must be investigated. One potential group of people to be examined is metabolically healthy obeses. The prevalence of obesity in the United States has increased by 75% since 1980 along with the acquisition of a western diet, and is associated with an increased incidence of cardiovascular disease, type 2 diabetes mellitus, hypertension, stroke, dyslipidemia, osteoarthritis, and some cancers (Burton and Foster, 1985; Ogden et al., 2007). However, ~10–30% of obese individuals are metabolically healthy and even have a lifelong health (van Vliet-Ostaptchouk et al., 2014). The physiological factors that characterized a metabolically healthy obese are decreased visceral and liver fat, number of macrophages in adipose tissue, mean adipocyte size, circulating C-reactive protein; while having an increase in serum adiponectin, and adipocyte insulin sensitivity (Klöting et al., 2010). The genetic background might play an important part in this scenario, as it has been observed that some ethnic groups at a higher body mass index (BMI) accumulate less liver fat, a factor that affects the metabolic outcome of the individual (Naukkarinen et al., 2014). A study revealed that liver fat content is higher among Japanese than non-Hispanic whites despite a lower mean BMI, and the difference becomes more robust with a small increase in BMI; this might explain why obesity-related complications in Asians occur at a lower BMI (Azuma et al., 2009).

In African Americans, high rates of fructose malabsorption have been associated with reduced liver fat (Walker et al., 2012). African-Americans also appear to be more resistant to hypertriglyceridemia (high blood levels of triglycerides) associated with insulin resistance (Guerrero et al., 2009). Geographical factors might be also involved; migrants from lower-to-higher chronic disease areas (i.e., Japaneses that migrate to the United States) acquire a higher risk of developing a chronic disease (Marmot et al., 1975). But even under a similar background, differences are observed. Naukkarinen et al. studied 16 Finnish pairs of identical twins in which one twin was obese and the other lean, they found that despite all twin pairs being of the same age, had similar age of onset of obesity and weight difference, half of the obese co-twins were metabolically as healthy as their lean co-twins while the other half of the obese co-twins exhibited a typical response to obesity, this was increased insulin production and resistance, dyslipidaemia, fatty liver, and higher blood pressure; they also observed that the one factor that best predicted the metabolic outcome was the level of liver fat (Naukkarinen et al., 2014).

It is now recognized that the gut microbiota can influence liver fat in the host, thus the microbiota might be one of the factors modulating the individual susceptibility to chronic disease. A study that observed an association of microbiota and liver fat accumulation demonstrated that gut microbiota directly induced NAFLD in mice. The authors performed fecal transplantations from mice that developed, or not, liver steatosis (responders and non-responders, respectively) during a 16 week period of high-fat diet (HFAD) to receiver mice. The responder-receiver mice developed a higher level of liver steatosis and had higher levels of branched-chain fatty acids from bacterial amino acid fermentation than non-responder-receiver mice (Newgard, 2012). Similarly, non-alcoholic steatohepatitis (NASH) (severe hepatic steatosis and liver inflammation) patients had an increase in ester compounds and endogenous alcohol most likely produced from bacterial metabolism compared to patients with simple steatosis (fatty liver) and healthy volunteers. It is worth mentioning that healthy subjects and obese non-NASH patients had similar blood-ethanol concentrations (Raman et al., 2013; Zhu et al., 2013; for a review see Boursier and Diehl, 2016), thus indicating that even under an obese state, non-NASH patients may harbor a microbiota whose functionality resembles the one on a healthy state. In addition, the administration of probiotics can exert a positive effect on liver fat accumulation, which will be mentioned later. One potential mechanism for liver fat accumulation is that bacteria can suppress the expression of a lipoprotein lipase inhibitor, the fasting-induced adipose factor (Fiaf), thus increasing the lipase activity in the gut and affecting the outflow of free fatty acids to the liver (Bäckhed et al., 2007). In order to advance our understanding of the factors influencing metabolic health during a western diet, it is important to explore the microbiome of metabolically healthy individuals following a western diet which stay healthy at an advanced age, such studies might reveal components of the microbiome that can counteract the accumulation of liver fat, protecting the host from further health outcomes.

## The western lifestyle and inflammation

Nowadays in the modern societies, an unbalanced diet, stress, and smoking can onset the inflammatory response daily, leading to a chronic low-grade systemic inflammation. Inflammation is the process through which the body limits pathogen invasion and controls tissue damage after injury. It is mediated by many soluble factors essential to signal immune cells to eliminate the aggressor and initiate tissue repair. Among these factors are secreted polypeptides called cytokines, which include tumor necrosis factor-α (TNF-α), interleukin-1β (IL-1β), interleukin-6 (IL-6), interleukin-10 (IL-10), IL-1 receptor antagonist (IL-1ra), and soluble TNF-α receptor (sTNF-R). TNF-α and IL-1β are pro-inflammatory cytokines, IL-6 has both anti- and pro-inflammatory properties, while IL-10, IL-1ra, and sTNF-R are anti-inflammatory cytokines. In acute inflammation, the levels of cytokines rapidly increase several fold and decrease when the infection is controlled or the injury is healed. However, acute inflammation does not always subside, and can become a chronic low-grade inflammation characterized by a two- to three-fold increase in the concentrations of cytokines and C-reactive protein, a molecule produced by the liver in response to inflammation (Petersen and Pedersen, 2005).

**Figure 1:**
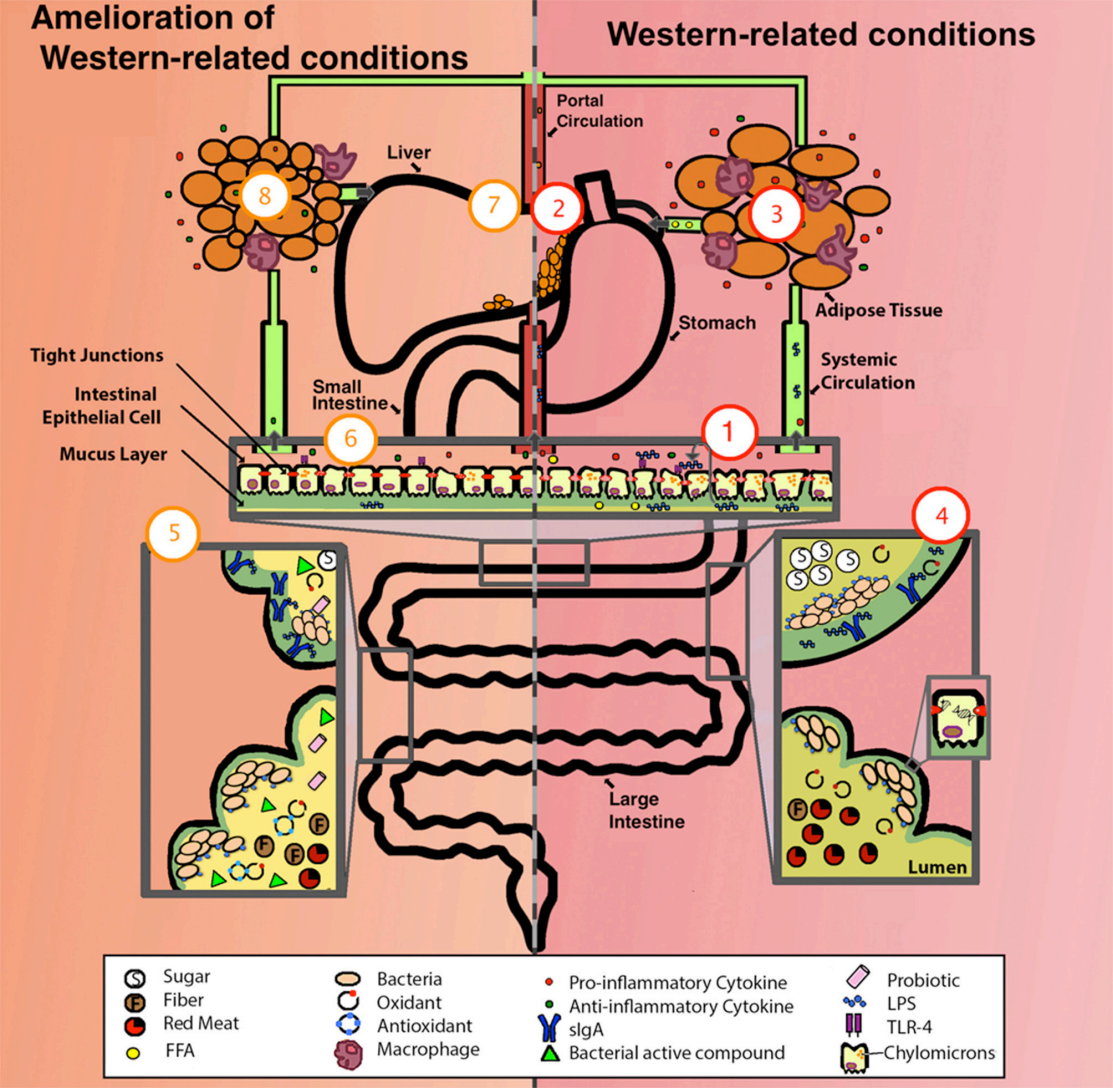
**Comparison of a system suffering from western-related conditions (right, in red) and a system with amelioration of western-related conditions (left, in orange)**. One aspect of a western lifestyle is the higher intake of ω-6 PUFA (depicted as FFA), this enhances the formation of chylomicrons allowing the translocation of LPS, these then activate basolateral TLR which initiates a pro-inflammatory response, one overall consequence is the alteration of the gut epithelium and its permeability (depicted as deteriorated epithelium and compromised tight junctions), exacerbating inflammation by allowing the translocation of more LPS, pro-inflammatory cytokines, FFA, among other luminal compounds (1). LPS/ pro-inflammatory cytokines/ FFA can enter portal and systemic circulation, one consequence is the alteration of fat metabolism, thus enhancing fat accumulation in liver (2), and in adipose tissue, adipocytes increase in size, FFA synthesis is enhanced (depicted as FFA in circulation), and an elevated pro-inflammatory state occurs (depicted as increased infiltration of macrophages and production of pro-inflammatory cytokines) (3). The western lifestyle includes higher intake of simple sugars and red-meat, lower intake of antioxidants (depicted as presence of oxidants), and sedentarism (depicted as low production of sIgA), some of the consequences are lower-capacity for antigen neutralization (depicted as LPS not bound to sIgA) and damage to the DNA of epithelial cells (depicted as DNA strand breakage) (4). For the amelioration of these conditions, a person can take different approaches, these include exercise (depicted as high production of sIgA), intake of dietary nutrients (i.e., polyphenols and ω-3 PUFAs) (depicted as antioxidants), probiotics, prebiotics, and SCFA (depicted as fiber, bacterial active compounds and probiotics) (5). Some of the effects of these approaches include the reestablishment of gut epithelium permeability and a decrease in LPS translocation, TLR activation, chylomicron formation, presence of LPS/cytokines/FFA in portal and systemic circulation (6), liver fat (7), adipocyte size, FFA synthesis, macrophage infiltration in adipose tissue (8), and an overall amelioration of the inflammatory state (depicted as a higher concentration of anti-inflammatory cytokines compared to pro-inflammatory cytokines [6 and 8]). FFA, free fatty acids. For more details see the text.

One way the western lifestyle can cause inflammation is by increasing the number of compounds and microbial products with inflammatory capability (Figure 1). Among these are: lipopolysaccharides (LPS) or endotoxins, D-lactate, acetaldehyde, hydrogen sulfide, toxic products of bacterial protein metabolism, and oxidative radicals, as described below. A role of antibiotics is also discussed.

### Lipopolysaccharides (LPS)

LPS are part of the outer membrane of Gram-negative bacteria and are one of the most important compounds that can induce a low-grade inflammation. LPS is bound by Toll like receptors (TLR) in cell surfaces, specifically TLR-4. Particularly in intestinal epithelial cells, these receptors mediate the inflammatory response triggering different mechanisms depending on its membrane location, apical or basolateral. Apical TLR are normally exposed to luminal antigens, including bacteria and their LPS, and their stimulation results in a homeostatic response and tolerance but not inflammation. In contrast, basolateral TLR are exposed to antigens only if these have crossed important epithelial barriers, and are potentially infectious. Therefore, basolateral TLR stimulation triggers the activation of the transcription factor NF-kβ, one of the most important mediators of the pro-inflammatory response (Wells et al., 2011). Extra-luminal LPS can also reach the bloodstream, and subsequently, bind TLR on the surface of other cells, like blood vessel, muscle, joint, adipose, and hepatic Kupffer cells. Their activation affects processes like insulin signaling, adipose tissue differentiation, lipogenesis, and it has been suggested that the interaction LPS-adipocyte–macrophage can amplify the low-grade inflammation to the level of influencing metabolic disorders (Muccioli et al., 2010). Thus, inflammation is a mechanism vital to set a prompt response to pathogens, however, LPS can onset a low-grade inflammatory response that may alter the metabolic status of the host by unknown molecular mechanisms (Nakarai et al., 2012). LPS are being increasingly associated with a number of conditions summarized in Table 1.

**Table 1:** **Conditions associated with high levels of plasmatic LPS**.

**Disease/Condition**	**Association with LPS**
Depression and neurodegenerative diseases	Peripheral inflammation can chronically activate brain microglia to produce elevated pro-inflammatory factors (Qin et al., 2007; Maes et al., 2013; Suffredini and Noveck, 2014).
Cardiovascular disease and atherosclerosis	Macrophages with a pro-inflammatory profile induced by TLR accumulate in blood vessel walls eventually forming a plaque (Wiedermann, 1999; Caesar et al., 2010).
Chronic fatigue syndrome	Serum levels of antibodies directed against LPS correlate to the level of fatigue (Maes and Leunis, 2008).
Cancer	LPS have been shown to increase the inflammatory activity of immune cells that generate oxidative radicals incrementing the chance of DNA damage in proliferating cells (Coussens and Werb, 2002), and they also increase the adhesiveness and metastatic capacity of cancer cells (Hsu et al., 2011).
Type 2 diabetes mellitus	LPS decreased insulin sensitivity in healthy subjects that had a reduced response to insulin 24 h after a LPS infusion protocol (Mehta et al., 2010).
Obesity	LPS are identified as a triggering factor since a 4-week treatment of LPS in mice resulted in a similar whole-body, liver, and adipose tissue weight gain as in a HFAD (Cani et al., 2007).
Autism	The higher the level of LPS, the worse the social interaction of the patient (Emanuele et al., 2010).
Systemic lupus erythematosus disease	LPS increase systemic nucleosome release due to an enhancement of apoptosis and a decrease in the clearance of apoptotic cells (Licht et al., 2001).
HIV-1	LPS lead to neurological dysfunctions since the increase of cytokine production affects the permeability of the blood-brain barrier allowing the trespassing of the virus into the brain (Dohgu and Banks, 2008).
Retinal pathologies	LPS are an underlying factor for their progression due to the sensitivity of the retinal pigment epithelium cells to inflammatory stress (Leung et al., 2009).
Autoimmune Joint Inflammation	An oral administration of LPS can exacerbate arthritis in animal models and antibiotics can suppress the recurrence of the disease (Yoshino et al., 1999).

Several behaviors associated with the western lifestyle can affect the levels of plasmatic LPS. Among these are sedentarism, smoking, stress, and an unhealthy diet. Lira et al. showed that sedentary people had higher levels of plasmatic LPS than highly trained people at rest (Lira et al., 2010). Pace et al. observed that cigarette smoke increased the expression of TLR-4 and LPS binding (Pace et al., 2008). Furthermore, it has been demonstrated that stress hormones stimulate the growth of LPS-containing bacteria such as *Yersinia enterocolitica* and *Escherichia coli*, and indeed, stress hormones achieved a 100,000-fold increase in viable *E. coli* in the cecum of mice within 24 h and promote the synthesis of an autoinducer of bacterial growth (Lyte and Ernst, 1992; Lyte et al., 1996; Lyte and Bailey, 1997; Freestone et al., 2008). Meanwhile, Cani et al. observed that a 4-week HFAD chronically increased plasma LPS concentration two to three times (Cani et al., 2007).

Among the factors that increase the abundance of plasmatic LPS, diet is the best studied. It is recognized that HFADs induce high levels of LPS in the blood through the stimulation of chylomicron (droplets of fat) formation in intestinal epithelial cells, this facilitates LPS transcellular transport across the gut epithelium and subsequently, LPS reach the bloodstream (Ghoshal et al., 2009). However, several investigations point that not every type of HFADs increases the concentration of plasmatic LPS. HFADs consisting of oils rich in ω-6 polyunsaturated fatty acids (ω-6 PUFA), like safflower oil, cause a markedly increase in the concentration of plasmatic LPS and pro-inflammatory cytokines compared to diets rich in coconut oil or fish oil, which instead are protective against a LPS challenge (Mascioli et al., 1988; Sadeghi et al., 1999). Meanwhile, a high-fructose diet (HFUD) promotes a more pronounced increase in plasmatic LPS concentration than diets rich in glucose. The mechanism for this is unknown, but evidence suggests that HFUD effects are related to the gut microbiota, since observations that oral non-absorbable antibiotics (antibiotics that act locally in the gut) can prevent the increase of plasmatic LPS, while the knockout of the LPS receptor TLR-4 greatly decreases lipid peroxidation, expression of TNF-α, and accumulation of fat in the liver that occurs in fructose-fed mice (Di Luccia et al., 2015). In humans, HFUD is also associated with NAFLD (Bergheim et al., 2008; Ouyang et al., 2008). The distinct effects of different fats and sugars might explain some of the variability of diet response among studies.

### Diet-dependent products of bacterial metabolism

Bacterial products of metabolism released in our gut depend heavily on diet, host secretions and digestive enzymes, local conditions of pH, oxygen, and hydrogen, gut transit time, and the composition and activity of the microbiota, among other factors (Salonen and de Vos, 2014). Undigested dietary residues that arrive to the large intestine are the main substrates of bacterial metabolism, along with diet-independent substrates like endogenous host secretions. Undigested carbohydrates are fermented mainly to short-chain fatty acids (SCFAs) (such as butyrate, acetate, and propionate) and gases (mainly carbon dioxide, hydrogen, and methane) (Flint et al., 2012). However, an excessive consumption of carbohydrates can also increase the concentration of toxic compounds derived from microbial metabolism, as it is the case of D-lactate, which is produced during carbohydrate fermentation by D-lactic acid bacteria. This compound inhibits the transport of L-lactate and pyruvate, both essential for mitochondrial energy production (Ling et al., 2012). Several conditions have been associated with high concentration of D-lactate, among these are chronic fatigue syndrome, diarrhea, short bowel syndrome, and diabetes (Uribarri et al., 1998; Ewaschuk et al., 2005; Sheedy et al., 2009). Another toxic compound that has been associated with the excessive consumption of carbohydrates and alcoholic drinks is acetaldehyde. This compound is produced by ethanol-oxidizing bacteria and yeast, and is formed during ethanol metabolism. When acetaldehyde is metabolized, oxidative radicals are generated, altering the permeability of the intestinal epithelium facilitating the translocations of luminal contents to the bloodstream (Atkinson and Rao, 2001). Acetaldehyde is also a known carcinogenic compound (Salaspuro, 1996; Wright et al., 1999).

While carbohydrates are fermented in the proximal colon, amino acids are fermented in the distal colon and this results in branched-chain fatty acids and potentially toxic metabolites such as ammonia, phenols, indoles, amines, TMAO, and volatile sulfur compounds (den Besten et al., 2013), some of which are associated with the increased incidence of colorectal cancer (Hughes et al., 2000; Russell et al., 2011) and atherosclerosis (Koeth et al., 2013) in high-red meat diets, fresh or processed. In the case of ammonia, higher levels of this compound in the blood can enter the brain and cause conditions like hepatic encephalopathy. Ammonia is a concern in subjects with chronic diseases in the thyroid gland, kidneys, lungs, and liver, and it is been increasingly associated with diabetes, extreme obesity (Bengmark, 2009), and tumor promotion (Hughes et al., 2000). Interestingly, the evidence suggests that white meat (poultry and fish) do not have the same detrimental effects of red meat. A possible explanation is the higher content of dietary haem in red meat, which will provide a source of iron for some proteins that can form toxic nitrosating agents from nitric oxide under anaerobic conditions (Wade and Castro, 1990).

Another toxic compound of bacterial protein or carbohydrate metabolism is hydrogen sulfide (H_2_S). This is produced by sulfate-reducing bacteria during the oxidation of a wide range of substrates found in the large intestine (Gibson et al., 1991). In western countries there is a high incidence of people with a sulfate-reducing bacteria, 50–70% compared to 10–20% of rural black Africans. The H_2_S produced by this group of bacteria can cause DNA damage in susceptible subjects with genetic predisposition that compromises DNA repair, as it is observed in patients suffering from ulcerative colitis and colorectal cancer (Attene-Ramos et al., 2006). The concentration of these toxic compounds of bacterial protein and carbohydrate metabolism in the intestinal lumen might be the result of interplay between the microbiota capacity to produce them and the host capacity to clear them up; in addition, the metabolic effect of these compounds would depend on the host susceptibility.

### Oxidative radicals

Oxidative radicals are normally produced in high concentrations during food digestion and are also generated during cigarette smoking. Ingestion of food with antioxidants can control the exposure to these compounds, consequently diets with low levels of antioxidants will not subside the constant oxidative stress that occur in the gut and lung epitheliums (van der Vaart et al., 2004; Bloomer and Fisher-Wellman, 2010; Caesar et al., 2010). Overnutrition also increases the oxidative stress in the endoplasmatic reticulum, this activates a mediator of inflammation normally inactive in the hypothalamus, the kinase IKKβ, which regulates NF-κβ through the phosphorylation of its inhibitor IkBα (Zhang et al., 2008). Oxidative stress also increases the activity of the PI 3-kinase and the myosin light chain kinase promoter that regulate the opening of the intestinal tight junction barrier. Thus, oxidative stress mediates the enlargement of the spaces in the gut epithelium allowing the translocation of normally non-invasive bacteria or their toxic products and components, which will induce the activation of NF-κβ perpetuating a vicious cycle of NF-κβ activation and impairment of the tight junction barrier (Sheth et al., 2003; Maes and Leunis, 2008).

In summary, the NF-κβ pathway that mediates inflammation can be activated by several cellular stresses, including LPS and compounds that generate cellular damage, like D-lactate, acetaldehyde, and H_2_S. Importantly, the activation of NF-κβ in parts of the body different from the gastrointestinal tract might eventually alter the permeability of the intestinal epithelium, facilitating the translocation of luminal materials, including LPS, which will exacerbate the low-grade inflammation state.

### Antibiotics

The use of antibiotics in the modern era, including the extensive and inappropriate use in humans and animals, has changed the gut microbiota and this has diverse health implications. Broad-spectrum antibiotics can impact the gut microbiota causing a dysbiosis (“a pathological imbalance in a microbial ecological niche” Jones et al., 2014) which can alter the microbiota capacity to prevent the colonization and growth of pathogens and pathobionts with inflammatory capability. Two meta-analyses, one in >56,000 patients with *C. difficile* infection and the other in >7000 inflammatory bowel disease (IBD) patients showed that antibiotics were a high risk factor for the development of these diseases (Furuya-Kanamori et al., 2014; Ungaro et al., 2014). Depending on the class of antibiotic, the dosage, time of administration, and other antibiotic-independent factors, like genetic predisposition, sex, diet, physical activity, disease, and environmental toxicants, antibiotics can exert effects on the weight (underweight and overweight states) and metabolic profile (pro-diabetic and anti-diabetic effect) of an individual (for a review see Cox and Blaser, 2015). Antibiotic use carries other risks, like the dissemination of bacterial resistant genes and the alteration of the well-established host-microbiota symbiosis through the eradication of important susceptible strains (Blaser and Falkow, 2009). Recently, Moeller et al. demonstrated the cospeciation of certain symbiotic bacterial strains with hominids, including humans (Moeller et al., 2016). This unique set of symbionts might provide beneficial health effects to the host and could be under selective pressure by the modern use of antibiotics.

## Approaches that counteract the western lifestyle

The approaches that can effectively counteract the effects of the western lifestyle are the ones that mitigate the translocation of LPS, prevent toxic microbial metabolism, and modulate the pro-inflammatory response and oxidative stress. Among these approaches are: exercise, dietary compounds, probiotics, prebiotics, and short chain fatty acids (SCFAs) (Figure 1 and Table 2), as described below.

**Table 2:** **Summary of beneficial factors under a western lifestyle**.

**Beneficial factor**	**Benefits**	**Benefited population**
Exercise	Exercise reduces pro-inflammatory state, liver fat, protects against insulin resistance, and increases the levels of SCFAs and sIgA (Schmitz et al., 2002; Starkie et al., 2003; Matsumoto et al., 2008; Nichol et al., 2008; Campos-Rodríguez et al., 2016).	Beneficial in the general population (its effects are under study for some conditions) (Gleeson et al., 2011).
Dietary polyphenols	Polyphenols function as antioxidants, strengthen intestinal barrier function, maintain beneficial bacterial strains, and prevent endotoxemia and the development of diabetes (Sies et al., 2005; He et al., 2012; Cowan et al., 2013; Anhê et al., 2015; Wang et al., 2016).	Beneficial in the general population (Manach et al., 2005).
ω-3 polyunsaturated fatty acids	ω-3 PUFA can reverse some of the inflammatory effects of ω-6 PUFA, like immune cell infiltration and NF-kβ activation, and enrich *Lactobacillus* spp. and *Bifidobacteria* spp. (Thompson and Spiller, 1995; Ghosh et al., 2013).	People with high ω-6 PUFA intake (Ghosh et al., 2013).
Prebiotics	Prebiotics increase abundance of *Bifidobacteria*, butyrate-producing and mucin-degrading bacteria. They have been observed to reduce body weight gain, dyslipidemia, inflammation, hypertension, and insulin resistance (Marcil et al., 2003; Tappenden et al., 2003; Galisteo et al., 2008; Delzenne et al., 2013; Kimura et al., 2013; Park et al., 2015).	Beneficial for the general population, as long as the individual has a microbiota with the capacity of degrading the prebiotic (Chambers et al., 2015).
SCFAs	SCFAs prevent weight gain, abdominal adiposity, liver fat, and reduced insulin resistance (Chambers et al., 2015).	These compounds have been observed to be beneficial in gastrointestinal disorders and overweight adults, but might be beneficial in other conditions not yet studied (Segain et al., 2000; Chambers et al., 2015).
**PROBIOTICS**		
*L. curvatus* HY7601 and *L. plantarum* KY1032	These strains prevent weight gain, and lower plasma glucose, insulin, triglycerides, oxidative stress levels, liver mass, and liver cholesterol (Park et al., 2013a,b).	People following a HFAD and HFUD (Park et al., 2013a,b).
*A. muciniphila*	This strain reduces plasmatic LPS, adiposity, insulin resistance, body weight, and hyperglycemia. It increases adipocyte differentiation and lipid oxidation, and prevents the thinning of the mucus layer (Everard et al., 2013).	People following a HFAD and/or with low abundance of mucin-degrading bacteria (Everard et al., 2013).
*B. uniformis* CECT 7771	This strain reduces total body weight gain, intestinal lipid absorption, liver fat, levels of cholesterol and triglycerides. It improves glucose metabolism, insulin and leptin sensitivity, and immune function. A Bacteroides-rich microbiota has been associated with reduced production of pro-atherosclerotic TMAO (Gauffin Cano et al., 2012).	People following a HFAD, and/or with a high inflammatory profile, and/or a high intake of red-meat (Gauffin Cano et al., 2012).
*L. reuteri* JBD30 l	This strain absorbs FFAs and increases fecal fat excretion (Chung et al., 2016).	People following a HFAD (Chung et al., 2016).
*L. fermentum* ME-3	This strain reduces post-prandial oxidative stress (Kullisaar et al., 2011).	People with low antioxidant intake (Kullisaar et al., 2011).
*L. acidophilus* strains NCFM and N-2	These strains lower the production of free amines and the activity of cecal bacterial ß-glucuronidase, nitro-reductase, and azoreductase enzymes (Goldin and Gorbach, 1984a).	People with a high red-meat intake (Goldin and Gorbach, 1984a).
*B. bifidum, L. plantarum* 8PA3, and *L. rhamnosus* GG	These strains improve liver function and lower alcohol-induced endotoxemia and hepatic steatosis (Kirpich et al., 2008; Wang et al., 2011).	People with high alcohol-intake (Kirpich et al., 2008; Wang et al., 2011).
*L. plantarum* 299v	This strain reduces systolic blood pressure, leptin, fibrinogen, IL-6, and monocytes adhesion to vein endothelial cells (Naruszewicz et al., 2002).	Heavy smokers (Naruszewicz et al., 2002).

### Exercise

Regular moderate doses of physical activity can ameliorate the effect of an LPS insult. In addition, it has been shown that exercise: controls the levels of pro-inflammatory cytokines, is associated with less liver fat (Starkie et al., 2003; Nichol et al., 2008), protects against insulin resistance (Schmitz et al., 2002), and increases the levels of SCFAs. The main SCFAs are butyrate, acetate and propionate, and these have anti-carcinogenic as well as anti-inflammatory properties and are essential for colon health (Matsumoto et al., 2012). Exercise can also modulate the microbiota, mice who exercised had lower intestinal and systemic bacterial loads than the group of sedentary mice, and had higher total and specific intestinal secretory immunoglobulin A (sIgA) which are the antibodies that control luminal antigens (Campos-Rodríguez et al., 2016).

### Dietary compounds

Dietary compounds can also be introduced to prevent the negative effects of a western lifestyle. Among these are: dietary polyphenols and ω-3 PUFAs. Polyphenols can be found in wine, cocoa, cranberry, grape, curcumin, propolis, coffee, and tea; they function as antioxidants (Sies et al., 2005), strengthen intestinal barrier function (Wang et al., 2016), prevent endotoxemia (presence of LPS in the blood), the loss of some beneficial bacterial strains, and the development of diabetes (He et al., 2012; Cowan et al., 2013; Anhê et al., 2015). The other compounds are ω-3 PUFA, which are found in fish and olive oil, their addition to a high ω-6 PUFA diet can reverse some of the inflammatory effects of ω-6 PUFA, like immune cell infiltration and NF-kβ activation (Ghosh et al., 2013). It is possible that the beneficial effects of some of these dietary compounds are exerted through the modulation of the microbiota. For example, the administration of cranberry extract and grape polyphenols is associated with an increased abundance of the beneficial genus *Akkermansia* even under a high sucrose and/or HFAD, while ω-3 PUFA have been shown to enrich *Lactobacillus* and *Bifidobacteria* (Thompson and Spiller, 1995; Ghosh et al., 2013; Anhê et al., 2015; Roopchand et al., 2015).

### Probiotics

A probiotic can be defined as “a live microorganism that, when administered in adequate amounts, confers a health benefit on the host” (FAO/WHO, 2002). Several recent studies have evaluated the benefits of probiotic supplementation in the absence of lifestyle changes (Doron and Gorbach, 2006). One example is the study of Park et al. who observed that mice following a HFAD for 8 weeks and supplemented with *Lactobacillus curvatus* HY7601 and *Lactobacillus plantarum* KY1032 for another 10 weeks gained 38% less weight than the unsupplemented controls (Park et al., 2013a). The same group also demonstrated that *L. curvatus* HY7601 and *L. plantarum* KY1032 at high (10^10^ cfu/d) or low dosage (10^9^ cfu/d) lowered plasma glucose, insulin, triglycerides, and oxidative stress levels in rodents fed a HFUD, while only at high doses lower liver mass and liver cholesterol were achieved (Park et al., 2013b).

The bacteria *Akkermansia muciniphila* and *Bacteroides uniformis* have also been evaluated under a HFAD. Everard et al. showed that *A. muciniphila* reduced plasma levels of LPS, adiposity, insulin resistance, body weight (without changing food intake), hyperglycemia, increased adipocyte differentiation and lipid oxidation. The supplementation of live cells of *A. muciniphila* also prevented the thinning of the mucus layer that occurred when mice were fed a HFAD (Everard et al., 2013). Meanwhile, Gauffin Cano et al. (2012) showed that an oral administration of *B. uniformis* CECT 7771 significantly reduced total body weight gain, liver fat, levels of cholesterol and triglycerides. Furthermore, *B. uniformis* CECT 7771 improved glucose metabolism, insulin and leptin sensitivity, and immune function of macrophages and dendritic cells. The authors also measured the number of fat micelles per enterocyte as an indicator of intestinal lipid absorption which contributes to adiposity, and *B. uniformis* CECT 7771 also achieved a significant reduction in this aspect (Gauffin Cano et al., 2012). It was also demonstrated, in meat-fed rats, that *Lactobacillus acidophilus* strains NCFM and N-2 promoted a significantly lower production of free amines (Goldin and Gorbach, 1984a) and lowered significantly, in rats and subjects, the activity of cecal bacterial ß-glucuronidase, nitro-reductase, and azoreductase enzymes which are responsible for the generation of potential precarcinogenic compounds (Goldin and Gorbach, 1984b).

Studies have not only evaluated probiotic effects under a particular nutritional environment but have examined their effect on the treatment of alcohol-drinking and smoking induced diseases. Several studies have shown an improvement of alcohol-induced liver injury in mice and human subjects. For example, Kirpich et al. performed a pilot study evaluating the effect of a 5-day probiotic supplementation consisting of *Bifidobacterium bifidum* and *L. plantarum* 8PA3 on 66 alcoholic individuals, the subjects under the probiotic treatment had significantly lower alanine aminotransferase (ALT) and aspartate aminotransferase (AST) activity than those treated with standard therapy (abstinence plus vitamins) (Kirpich et al., 2008). Then again, the same group demonstrated that a *Lactobacillus rhamnosus* GG supplementation during the last 2-weeks of a 8-week diet containing 5% alcohol significantly improved liver function and reduced alcohol-induced endotoxemia, and hepatic steatosis in mice (Wang et al., 2011). Meanwhile, Naruszewicz et al. determined that the administration of *L. plantarum* 299v to heavy smokers for 6 weeks with no changes in lifestyle led to the significant reduction in systolic blood pressure, leptin, fibrinogen, IL-6, and monocytes adhesion to vein endothelial cells, thus reducing their risk of cardiovascular disease (Naruszewicz et al., 2002).

### Prebiotics and SCFAs

Prebiotics are non-digestible fiber that promotes the growth of beneficial microorganisms in the gastrointestinal tract. Some examples are: inulin, fructooligosaccharides, resistant starch, pectin, among others. These are metabolized to SCFAs, mainly propionate, acetate and butyrate, which as mentioned earlier exert many beneficial health outcomes. SCFAs activate the SCFA receptor GPR43 that reduces insulin sensitivity in adipose tissue and hence its fat accumulation, thereby reducing the uptake, synthesis, and oxidation of toxic fatty acids in other tissues (Kimura et al., 2013; Park et al., 2015). They also increase proliferation and inhibit apoptosis of intestinal cells (Tappenden et al., 2003), hinders intestinal secretion of chylomicron into the circulation (Marcil et al., 2003), and limits inflammation perhaps through inhibition of the NF-κβ pathway (Delzenne et al., 2013). Galisteo et al. analyzed several studies that showed that prebiotics reduce all the abnormalities clustered in the metabolic syndrome, including: body weight gain, dyslipidemia, inflammation, hypertension, and insulin resistance (Galisteo et al., 2008).

One drawback of prebiotics is that they can cause intestinal tract discomfort in individuals with limited microbial capacity to ferment the prebiotic. Thus, novel approaches to deliver the benefits of prebiotics have been developed. Chambers et al. (2015) designed an improved prebiotic compound linked to a SCFA, propionate, which exploits the benefits of prebiotics while reducing the amount to be administrated. SCFAs have many health benefits, but if they are supplemented orally, they will be absorbed in the upper part of the small intestine where their benefits are limited. In contrast, this new compound ensures the delivery of the SCFA directly to the colon and at the same time, reduces the patient complains about prebiotics (e.g., gas production and bloating). Only 10 g of the inulin-propionate ester achieved a 2.5-fold increase in colonic propionate, this in consequence, prevented weight gain, abdominal adiposity, liver fat, and reduced insulin resistance significantly more than in the prebiotic-only control group (Frost et al., 2013; Chambers et al., 2015).

Probiotics and prebiotics are already been used clinically for the improvement of fatty liver (Ma et al., 2013), minimal hepatic encephalopathy (Liu et al., 2004), diabetes (Asemi et al., 2014), abdominal adiposity (Kadooka et al., 2010), chronic fatigue syndrome (Maes and Leunis, 2008), diarrhea, and *Clostridium difficile* disease (McFarland, 2006), among others. Their supplementation is one alternative that is simple, safe, and that improves several health parameters simultaneously.

## Mechanism-based screening strategies for the identification of beneficial strains

There is great interest in developing commercial probiotic formulations that include new beneficial strains. Thus, several studies focus on different screening strategies to find promising strains that can favorably shape host pathways. These strains can act directly or indirectly on the cells of the immune system, epithelial cells, adipocytes, beta pancreatic cells, and can also control pathobionts. For instance, Gauffin Cano et al. screened for the immunomodulation capabilities among different strains of *Bacteroides* spp., they carefully selected for a specific strain that had the lowest inflammatory potential on macrophages *in vitro*, specifically, low TNF-α and high IL-10 production (Gauffin Cano et al., 2012). Poutahidis et al. (2013) also demonstrated that *L. reuteri* protected the host from obesity through an immunomodulatory mechanism, specifically, *L. reuteri* had an effect on the IL-10-dependent function of CD4+ T cells. Interestingly, the researchers could replicate the phenotype of the probiotic-supplemented mice in naïve recipient rodents by transferring only the purified CD4+ T cells (Poutahidis et al., 2013). Meanwhile, Ito et al. screened the inhibitory activity of 49 lactic bacterial strains on lipid peroxidation *in vivo* and *in vitro* (Ito et al., 2003). While Kullisaar et al. measured the capacity of *L. fermentum* ME-3 to reduce oxidative stress, blood triglyceride levels, and lipoprotein status post-prandially (2 h after a meal) in a randomized double-blind placebo-controlled study with 100 healthy subjects (Kullisaar et al., 2011). Lastly, Chung et al. (2016) screened for a FFAs-absorbing strain, *L. reuteri* JBD30 l, in a fecal sample of a healthy lean subject. The administration of this strain to experimental animals and human subjects under a clinical trial lowered the concentration of FFAs in the fluid of the small intestine thus increasing fecal fat excretion, the efficacy was comparable to the one obtained for orlistat, a FDA-approved pharmaceutical that also increases the content of fat in feces (Chung et al., 2016).

There are other reported mechanisms that can guide screening studies, among these are the increment in the expression of lectins against Gram-positive bacteria, e.g., *A. muciniphila* produces RegIIIγ (Everard et al., 2013); the inhibition of T cell activation, e.g., *S. boulardii* produces a <3 kDa protein that has this effect (Thomas et al., 2011); production of phosphatases that can dephosphorylate LPS, as it has been observed also in *S. boulardii* (Buts et al., 2006); upregulation of the expression of cytoprotective heat shock proteins that increase the protection against oxidative damage and gut barrier loss in intestinal cells, e.g., *Bacillus subtilis* produces a quorum-sensing signal molecule, the competence- and sporulation-stimulating factor, which induces the heat shock protein Hsp27 (Fujiya et al., 2007); inhibition of the hydrogen peroxide-induced epithelial barrier disruption, e.g., *L. rhamnosus* GG produces two soluble proteins, p40 and p75, that control this aspect (Seth et al., 2008); inhibition of NF-kβ pathway (Petrof et al., 2004); and enhancement of SCFA production (Kimura et al., 2013; Park et al., 2015).

## Conclusions

The western lifestyle causes the overproduction of inflammation signals and underprovides the means to block them, driving the body into a chronic low inflammation state. To avoid some negative consequences, people can introduce light exercise, simple dietary compounds, probiotics, prebiotics and/or SCFAs into their daily routine. Interestingly, several recent studies have proved that the effects of probiotics and prebiotics can even be exploited under a HFAD and smoking conditions, providing a way to extend the health of a person with a western lifestyle. The presence of probiotics in dairy products has made them well accepted and recognized by their health benefits on the gastrointestinal tract, and given that the clinical evidence points that they also have benefits on the lipid and glucose metabolism, gut permeability, mood, and immune system, it is foresighted that this field will keep introducing new probiotic strains to the market, perhaps specific formulations depending on the desired benefit. We proposed that for the advancement of this field, it is important to understand if there is a microbiological component that is extending the health of asymptomatic lean, overweight and obese people following a western diet, and the factors that increase the fitness of these strains in the western microbiome. Ultimately, considering that in western countries the most prevalent diseases are inflammatory in nature, it will be important that in the near future, inflammation markers would be routinely screened in the clinical setup and anti-inflammatory probiotics administered as an alternative preventive measure.

## Author contributions

GR conceived the work, GR, AC, AR, and FL wrote the paper and revised it critically.

## Funding

Research supported by Direction of Research (DIN), from Universidad de La Sabana (MED-198-2015), GR is supported by a grant from Colciencias, 647 2015. AR is supported by proyecto FAPA, P14.160422.001, Universidad de los Andes.

### Conflict of interest statement

The authors declare that the research was conducted in the absence of any commercial or financial relationships that could be construed as a potential conflict of interest.

## Abbreviations

BMI: Body mass index
HFAD: high-fat diet
HFUD: high-fructose diet
H_2_S: hydrogen sulfide
IL-1ra: IL-1 receptor antagonist
IL-10: interleukin-10
IL-1β: interleukin-1β
IL-6: interleukin-6
IBD: inflammatory bowel disease
IBS: irritable bowel syndrome
LPS: lipopolysaccharides
NASH: Non-alcoholic steatohepatitis
NF-κβ: Nuclear Factor kappa beta
SCFA: short chain fatty acid
sTNF-R: soluble TNF-α receptor
TLR: Toll like receptors
TNF-α: tumor necrosis factor-α
TMAO: trimethylamine-N-oxide
ω-3 PUFAs: ω-3 polyunsaturated fatty acids
ω-6 PUFA: ω-6 polyunsaturated fatty acids.
